# Mangiferin Rich Products from *Aphloia theiformis* (Vahl) Benn Leaves: Extraction, Fractionation, Phytochemical Characterization, and Antioxidant Properties

**DOI:** 10.3390/molecules25092081

**Published:** 2020-04-29

**Authors:** Dovilė Grauzdytė, Audrius Pukalskas, Chaker El Kalamouni, Petras Rimantas Venskutonis

**Affiliations:** 1 Department of Food Science and Technology, Kaunas University of Technology, LT-50254 Kaunas, Lithuania; dovile.grauzdyte@ktu.lt (D.G.); audrius.pukalskas@ktu.lt (A.P.); 2 UM 134 Processus Infectieux en Milieu Insulaire Tropical (PIMIT), INSERM U1187, CNRS UMR9192, IRD UMR249, Plateforme Technologique CYROI, Université de la Réunion, 97490 Sainte Clotilde, France; chaker.el-kalamouni@univ-reunion.fr

**Keywords:** antioxidant capacity, mangiferin, tormentic acids, extraction, fractionation

## Abstract

*Aphloia theiformis* is traditionally used in Mauritius, Madagascar, and Reunion Island for treating several diseases. In this study, various extraction solvents and schemes were applied for the recovery of antioxidant rich fractions from the leaves of *A. theiformis*. The products were evaluated for their antioxidant capacity using well known in vitro assays. Major compounds were characterized by UPLC–QTOF–MS. Hydrophilic extracts of *A. theiformis* demonstrated strong antioxidant properties, which are comparable with the synthetic antioxidant Trolox. UPLC analysis confirmed mangiferin as the main secondary metabolite of *A. theiformis*. Tormentic and hydroxytormentic acids as well as their isomers were also abundant in *A. theiformis* extracts and fractions, while their amounts were determined for the first time. The most potential extract was further separated into the fractions by liquid-liquid extraction and by precipitation at low temperature. Antioxidant capacity and composition of secondary metabolites of derived fractions were determined. Some of the fractions possessed remarkable antioxidant capacity, comparable to pure mangiferin. The results obtained reveal high potential of *A. theiformis* for recovery of natural antioxidants and other bioactive phytochemicals, particularly mangiferin.

## 1. Introduction

Natural products have been used in traditional medicine, foods, and cosmetics since ancient times. Plants biosynthesize thousands of various secondary metabolism products, among them valuable phytochemicals possessing antioxidant, antimicrobial, and many other health beneficial properties [1,2]. Considering vast biodiversity in the Plant Kingdom, bioguided investigation and valorization of less studied species remain an important topic for modern biosciences, particularly in order to discover new valuable ingredients and natural molecules for foods, nutraceuticals, pharmaceuticals, and cosmeceuticals.

Recovery, separation, fractionation, and purification of plant bioactive constituents are very important steps in the multistep process of bioguided assay of new phytochemical ingredients. Various extraction techniques, solvents, and schemes have been used for the extraction of bioactive constituents. The yield, recovery rate of target compounds, and bioactivity of extracts highly depend on the applied extraction method, therefore, its selection and evaluation is very important for the preparation of natural products [2,3]. Traditional solid-liquid extraction methods such as maceration and Soxhlet extraction are usually time consuming and require large amounts of solvents. Therefore, various more innovative extraction techniques including pressurized liquid extraction (PLE), supercritical fluid extraction (SFE), ultrasound assisted extraction (UAE), microwave assistant extraction (MAE), and others have gained popularity during the last decades. For example, PLE is an automated, time and solvents saving method, which enables the performance of fast and efficient extraction at subcritical solvent parameters [4], while SFE with CO_2_ produces solvent-free extracts at moderate temperatures reducing the risks of thermal degradation of target compounds without using hazardous organic solvents [5,6]. It is particularly attractive in developing green chemistry requirements fulfilling extraction processes.

Crude extracts usually require further fractionation for separating the substances with higher amounts of bioactive compounds from the complex mixtures, which are isolated from the plants using various solvents. However, it should be noted that in some cases the mixture of compounds may display better bioactivities than a single purified active constituent due to the synergistic effects. For instance, it was reported [7] that anti-tumour activity of isolated phytochemicals was weaker compared to their specific combination: the mixture of bioactive phytochemicals significantly inhibited cell proliferation, migration, and invasion in breast cancer cell lines, while individual constituents present in the applied mixture were less active.

*Aphloia theiformis* is a medicinal plant used in ethnomedicine for different purposes in the Indian Ocean islands and the East Africa region. Previously included in the Flacourtiaceae family, *A. theiformis* is currently the only one species of the Aphloiaceae family [8]. This plant has been used in traditional medicine for the treatment of dysentery, malaria, fever [9], cataract, and diabetes [10]. Previous studies have also reported the antiviral [11], antidiabetic [12], antioxidant [9], antimicrobial [13], and photoprotective [8] activities of *A. theiformis* leaves. The active ingredient in this plant appears to be a xanthonoid class compound mangiferin. Recently, mangiferin and its derivatives are used in cosmetic, food, and pharmaceutical industries: 115 patents focusing on therapeutic or cosmetic application of mangiferin, its derivatives and/or formulations had been registered until 2013 [14]. Mangiferin, due to its complex structure, is quite difficult to synthesize chemically, therefore extracting mangiferin from natural sources remains as the best possible alternative for its production [15].

Previous studies have already investigated *A. theiformis* for its cytotoxicity, antiviral, antidiabetic, antioxidant activities, and chemical profile [11,16,17]. However to the best of our knowledge, systematic studies on the extraction, fractionation, and evaluation of *A. theiformis* antioxidant activity and composition of secondary metabolites have not been performed previously. To fill this gap, the main aim of the present work was to test traditional (also called calventional) and innovative extraction methods with various polarity solvents, using single and consecutive extraction schemes for evaluating yield, secondary metabolites composition, and antioxidant potential of *A. theiformis*. Additionally, the extract with the best antioxidant capacity was further fractionated in order to determine the most powerful antioxidant products.

## 2. Results and Discussion

### 2.1. Extract Yields

**Figure 1:**
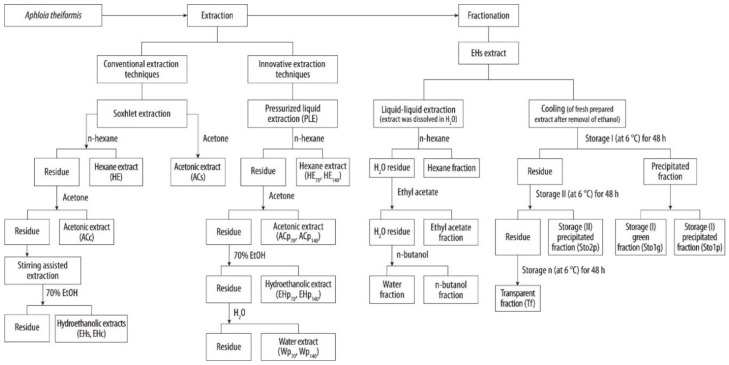
Schematic representation of extraction and fractionation procedure of *A. theiformis* leaves.

Botanicals are very complex biological structures composed of various groups of compounds, therefore proper selection and elaboration of extraction schemes is an important task for recovering the products of desirable properties and composition [18]. The extractability of phenolic compounds from the plant matrices depends on several factors including solvent properties and volume, pH, temperature, pressure, the number of extraction steps, and others [19]. In this study, single step and consecutive extractions with the increasing polarity aprotic and protic solvents were chosen for the isolation of lipophilic and hydrophilic extracts from *A. theiformis* leaves. In addition, traditional and high pressure (PLE) extraction techniques were compared (Table 1). The flowchart of preparation of extracts and fractions of *A. theiformis* is shown in Figure 1.

Nonpolar and widely used in lipid extraction *n*-hexane (relative polarity 0.09) gave the lowest extraction yields, however, the yield remarkably increased (by 37% compared to conventional extraction) when PLE was performed at 140 °C. Higher polarity aprotic solvent acetone (relative polarity 0.355) yielded remarkably higher amounts of extracts both from the initial plant material and from the residues after hexane extraction. It is interesting noting that the yields in PLE with acetone were lower than in conventional consecutive extraction. Reduced dielectric constant of acetone at high temperature might be one of the reasons of this finding. Stirring assisted extraction of the initial plant materials with 70% ethanol gave the highest yield of extract (34.07% ± 0.66%). The yields during consecutive extractions were measured for the dry weight of residues (DWR) and also estimated for the dry weight of the initial plant material (DWP). The total extract yield in consecutive extraction was from 37.1% (conventional method) to 45.4% (PLE at 140 °C) DWP. However, it should be noted that water extraction was used as the final step only in PLE and extraction yields from the three extraction steps (excluding water) were not considerably different between conventional and PLE methods. Higher PLE temperature gave only slight increase in the total yield. It was reported [2] that PLE yields from *Solidago virgaurea* leaves at 140 °C were almost two-fold higher compared with those at 70 °C. Regarding this finding, PLE extraction was performed at the same temperatures, however in the case of *A. theiformis* PLE, temperature did not have such a significant effect on extract yield.

### 2.2. Effect of Extraction Methods on the Antioxidant Capacity of Extracts

The experts in the area of the in vitro evaluation of antioxidant potential concluded that, due the complex nature of antioxidant phytochemicals and differences in reaction mechanisms, antioxidant properties of natural products may be evaluated more accurately by using several assays [20]. Following this recommendation, antioxidant properties of *A. theiformis* extracts were assessed by using four assays (Table 1). Determination of total phenolic content (TPC) with Folin–Ciocalteu’s reagent is based on a single electron transfer and therefore can also be assigned to antioxidant capacity assays. In addition, antioxidant capacity values were measured in different extracts and expressed not only in dry weight of extract (DWE) but also recalculated to dry weight of plant material (DWP). Both values are practically important as they show the antioxidant potential of the products obtained and evaluate the effectiveness of recovery of antioxidants from the dry plant material, respectively. Antioxidant capacity of lipophilic hexane extracts was not measured because this study focused on higher polarity polyphenolic extracts.

It is obvious that extract antioxidant capacity was highly dependent on extraction solvents and methods (Table 1). The TPC values of extracts were from 7.59 ± 0.55 (Wp_140_) to 330 ± 11.9 (EHs) mg GAE/g DWE. The radical scavenging capacity of *A. theiformis* extracts were from 1395 ± 111 (Wp_140_) to 3267 ± 81.5 (EHs) µmol TE/g DWE in 2,2-Diphenyl-1-picryhydrazyl hydrate stable radical (DPPH^•^) scavenging assay, while Trolox equivalent antioxidant capacity values in 2,2-azino-bis(3-ethyl-benzothiazoline-6-sulfonic acid) diammonium salt (ABTS)^•+^ decolourization assay ranged from 107 ± 2.89 (Wp_140_) to 5091 ± 118 (ACc) µmol TE/g DWE. The ability of extracts to reduce ferric ions to their respective lower valency ferrous state, as determined by the Ferric Reducing Antioxidant Power (FRAP) method, varied from 290 ± 26.3 (Wp_140_) to 4473 ± 129 (EHs) µmol TE/g DWE. Consequently, the antioxidant capacity of the most potential extracts was very strong, i.e., equivalent to 0.82–1.27 g of Trolox in 1 g of extract. Hydroethanolic extracts were the strongest antioxidants; acetone extracts were also strong antioxidants, however due to their low yield, 70% ethanol was the most suitable solvent for the best recovery of antioxidants from *A. theiformis*. It can be observed from the recalculated values to DWP, which in case of using ethanol–water mixture, were several times higher than in case of extracting plant material with acetone and water.

Comparing different extraction schemes, it may be observed, that the sum of recovered antioxidants was almost similar in consecutive extraction and PLE at 70 °C, while PLE at 140 °C gave lower values of antioxidants recovery. It may be assumed that some sensitive to heat antioxidants degraded at increased temperature. On the other hand, *n*-hexane due to the increased extraction effectiveness could also extract some antioxidants: hexane extract yield at 140 °C was higher by 45% than at 70 °C. Furthermore, antioxidant recovery with 70% ethanol using single step extraction by stirring was similar or even higher than the recovery of antioxidants obtained using consecutive extraction. For instance, 70% ethanol recovered 112 mg of phenolic compounds from 1 g of plant material. However, application of consecutive extraction schemes with different polarity solvents enables to achieve preliminary pre-fractionation of botanical bioactive compounds [21]. For instance, it was reported that the extracts isolated from *A. theiformis* with pressurized low polarity ethyl acetate (relative polarity 0.228) were very weak antioxidants in the all applied assays [22]. Other advantages of PLE are faster process, convenience and lower volumes of used solvents. 

### 2.3. Fractionation of the Crude Hydroethanolic Extract and Characterisation of Obtained Fractions

**Figure 2:**
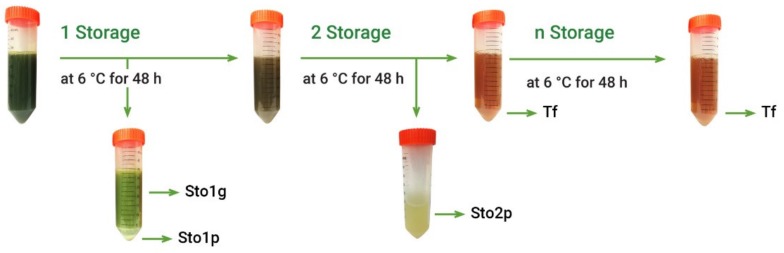
Fractionation scheme of hydroethanolic extract (EHs) of *A. theiformis*. Sto1g—green fraction (storage 1); Sto1p—precipitated fraction (storage 1); Sto2p—precipitated fraction (storage 2); Tf—transparent fraction.

Crude extracts usually contain large diversity of various components, while only some of them may demonstrate antioxidant properties, the others being neutral or even pro-oxidant [23]. Further separation step included simple fractionation of *A. theiformis* hydroethanolic extract by liquid–liquid extraction. Considering that the main plant phytochemical mangiferin is prone to precipitate at ambient and lower temperatures [8], freshly prepared hydroethanolic extract (EHs) was fractionated by cooling at 6 °C (Figure 2).

Among the solvent-partitioned fractions of *A. theiformis* EHs extract, the *n*-butanol (48.0%) showed the highest yield followed by the water (17.7%), ethyl acetate (17.0%), and hexane (14.2%) fractions (Table 2). The yield of fractions obtained by cooling varied markedly among fractionation procedures due to the difficulties in controlling precipitation process. Nevertheless, it could be observed that 36.3% of substances of crude EHs extract are prone to precipitate at low temperature.

The TPC and antioxidant capacity values of the fractions are listed in Table 2 and compared to pure mangiferin. In general, some fractions exhibited remarkably larger antioxidant capacities than the crude EHs extract. A similar tendency was also observed in the previous study [24], where the antioxidant potential of some fractions from peanut skin during the purification process increased relative to that of the crude extract. Among the fractions obtained by liquid–liquid extraction, *n*-butanol fraction had the highest antioxidant capacity followed by ethyl acetate and water fractions, while Sto2p fraction showed the highest antioxidant capacity among the fractions obtained by precipitation at low temperature. The most active fractions also had the highest TPC. Antioxidant capacities of hexane, dichlormethane, ethyl acetate, *n*-butanol and water fractions of crude methanol and aqueous extracts of *A. theiformis* were reported previoulsy [12]: ethyl acetate and *n*-butanol fractions demonstrated the most significant antioxidant properties. Furthermore, the crude methanol extract as well as ethyl acetate and *n*-butanol fractions proved to be potent inhibitors of α-amylase, α-glucosidase, and pancreatic lipase and contained the highest amounts of phytochemicals [12]. As indicated in Table 2, mangiferin is very strong antioxidant; therefore it may be considered to be responsible for the overal high antioxidant capacity of extracts. It is interesting noting that Sto2p fraction had significantly higher effects towards DPPH^•^ and ABTS^•+^ radicals than mangiferin, which suggests that other constituents present together with mangiferin may act synergistically in contributing to the high antioxidant capacity of *A. theiformis* extracts.

### 2.4. Characterization of Secondary Metabolites by Chromatography–Mass Spectrometry

**Figure 3:**
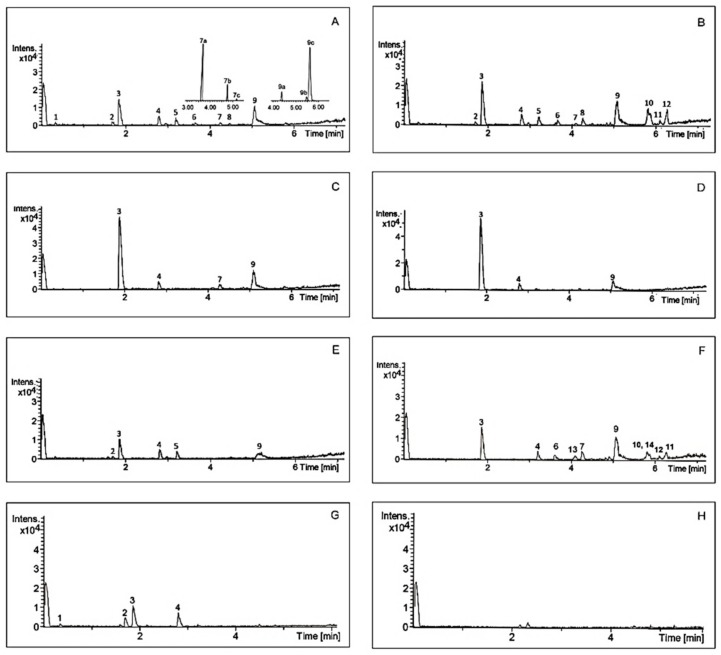
Chromatograms of *A. theiformis* EHs extract (**A**) and its fractions (**B**–**H**) obtained by UPLC-QTOF-MS. Sto1g (**B**), Sto1p (**C**), Sto2p (**D**), Tf (**E**), *n*-butanol (**F**), ethyl acetate (**G**), and water (**H**). For peak numbers see Table 3.

Analysis of chemical composition confirmed that *A. theiformis* is rich in mangiferin and some saponins. Typical chromatograms of *A. theiformis* EHs extract and its fractions are presented in Figure 3, while chemical profile is summarised in Table 3. Two constituents of *A. theiformis* belonging to triterpenoid saponins were identified as tormentic (TA) and hydroxytormentic (HTA) acids. Their presence was confirmed by comparing accurate masses and chromatographic retention times with reference standards.

Though mangiferin, TA, and HTA were previously identified in *A. theiformis*, their concentrations have not been determined. To fill this gap these compounds were quantified by UPLC-TQ-S using external standards. Their concentrations in different extracts and fractions are provided in Table 4 and Table 5. The recovery of the major quantitatively compound mangiferin was in the range from 1.14 to 35.6 mg/g DWP. For comparison, mangiferin content in other important source, mango tree leaves, was reported to be 12.94 mg/g DWP [25]. Another study determined mangiferin content in different fruit tissues (peel, pulp, and seed kernel) of Chinese mango cultivars and it was in the range from 0.002 to 7.49 mg/g DW, the highest mangiferin content was found in the fruit peel [26]. It should be taken into account that the amount of mangiferin and other bioconstituents belongs to the extraction conditions and solvents used. In our case, the highest recovery of mangiferin was obtained by hydroethanolic extraction (22.4–35.6 mg/g DWP), which confirms that *A. theiformis* is a rich source of mangiferin. Among purified fractions, Sto1p and Sto2p fractions had the highest amounts of mangiferin followed by *n*-butanol fraction, therefore it is not surprising that these fractions demonstrated the highest antioxidant capacity.

As presented in Figure 3A TA and HTA may have some isomers with the molecular weights of 487.3430 g/mol (**7a**, **7c**) and 503.3377 g/mol (**9a**, **9c**), respectively. It should be pointed out that these isomers are reported in *A. theiformis* for the first time. Due to the high structural similarity to HTA and TA, these isomers were quantified using the same calibration curves and their amounts were expressed as TA and HTA equivalents. The amount of HTA among extracts ranged from 0.04 to 0.43 mg/g DWP, the amount of **7a** isomer was quite similar to the amount of HTA. Although TA was previously isolated and identified in *A. theiformis*, however, our study showed that the amount of this compound was very small, only 0.02–0.05 mg/g DWP. Considerably higher amounts (0.05–0.40 mg/g DWP) of TA isomer with retention time of 5.74 min (**9c**) were recovered. This isomer was also dominant in the purified fractions. Ethyl acetate, Sto1p and Sto2p fractions had the highest amounts of this isomer, from 8.30 to 9.98 mg/g DWF.

TA isomer **9a** and HTA isomer **7c** were also observed in the extracts, however their amounts were rather low, 0.01–0.32 mg/g DWE. It is worth noting that TA, HTA and their isomers were more abundant in acetone extracts than in hydroethanolic or water extracts. Moreover, acetone extracts obtained by PLE had higher amount of TA and HTA than acetone extracts obtained by Soxhlet extraction. Nevertheless, the 70% ethanol, due to the high yields of hydroethanolic extracts, was the most suitable solvent for recovery of mangiferin, TA and HTA.

In the previous study on the chemical characterization of *A. theiformis*, three triterpene glucosides (C_36_H_58_O_10_, C_36_H_58_O_11_, and C_36_H_58_O_11_) were isolated and identified as TA ester glucoside, 23-HTA ester glucoside, and 6β-HTA ester glucoside, respectively [17]. In our study, these compounds were not found, however some other high molecular mass compounds with molecular formula C_37_H_60_O_14_, C_37_H_60_O_13_, and C_37_H_60_O_12_ were detected.

The MS/MS fragmentation of C_37_H_60_O_12_ gave two fragment ions with *m*/*z* 649.3949 and 487.3427. The ion with *m*/*z* 487.3427 fits TA ion formula, therefore, this compound was tentatively assigned to TA derivative. Previous study also reported that this fragment is characteristic to TA derivatives, e.g., TA 3fl-O-a-L-rhamnopyranoside [27]. MS/MS fragment ion with *m*/*z* 503.3371 fitting HTA ion formula, suggests that C_37_H_60_O_13_ compound may be a HTA derivative.

Some constituents were not identified due to the lack of spectral information; further purification steps and NMR analysis would be required for elucidating their structures. Nevertheless, the identified and quantified compounds, due to various previously reported biological activities such as antioxidant, anticancer, anti-inflammatory, and cytoprotective [28,29,30,31], can play a crucial role in health benefits of *A. theiformis*. In addition, it may be mentioned that tannins and flavonoids were also reported in *A. theiformis* [32].

### 2.5. Correlation Between Different Values

**Figure 4:**
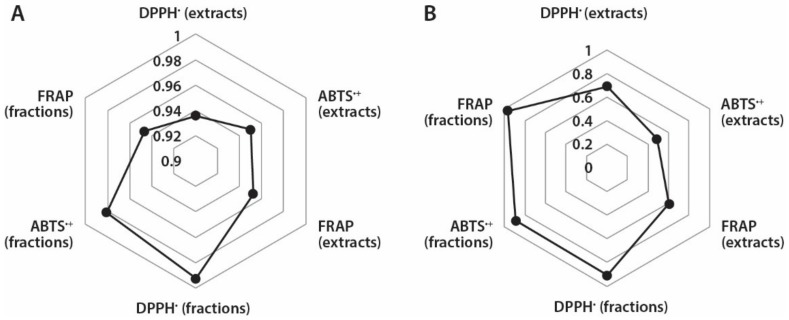
Correlation coefficients between: (**A**) TPC and different antioxidant measurement assays and (**B**) mangiferin content and different antioxidant measurement assays in extracts and fractions.

It is known that the correlation between antioxidant capacity values determined by different methods depend both on the specificity of the applied assay and phytochemical composition of tested plant material [33]. Therefore, it was interesting to assess if there are correlations between TPC and different antioxidant assays used in this study (Figure 4A). Based on the calculated correlation coefficients, strong correlations exist between TPC and different antioxidant capacity assays of *A. theiformis* extracts (0.936–0.952) and fractions (0.947–0.993). The strongest correlation occurred between the TPC and FRAP values, which were measured for extracts, while in case of fractions the strongest correlation was observed between TPC and DPPH^•^ scavenging values. It is important to note that the antioxidant capacity values expressed in DWE were used for calculations.

Mangiferin is the most important secondary metabolite in *A. theiformis* and therefore it was also interesting to determine if the correlations exist between mangiferin content and antioxidant capacity of extracts and fractions (Figure 4B). Strong correlations (0.892–0.969) were observed between mangiferin content and antioxidant capacity of fractions in DPPH^•^, ABTS^•+^, and FRAP assays, while correlation coefficients of extracts were quite weak, 0.487–0.689. In case of extracts the strongest correlation was observed between mangiferin content and DPPH^•^ scavenging assay (of extracts), and mangiferin content and FRAP assay (of fractions). These findings reveal that mangiferin was responsible for the high antioxidant capacity of fractions, while the absence of strong correlations of extracts indicated that the presence of other compounds in extracts could also contribute to their antioxidant properties. 

## 3. Materials and Methods

### 3.1. Plant Material

The leaves of *Aphloia theiformis* (Vahl) Benn were collected in Reunion Island (55°3′ E and 21°5′ S). The plants were dried at 37 °C during 2 days. The voucher specimens JF874 were deposited in the herbarium of the University of Reunion and identified by Professor Dominique Strasberg. Before extraction the plants were ground in the laboratory mill Retsch ZM200 (Retch GmbH, Haan, Germany) using a 1 mm sieve.

### 3.2. Chemicals and Reagents

All chemicals and reagents used in this study were of analytical or HPLC grade. 2,2-Diphenyl-1-picryhydrazyl hydrate stable radical (DPPH^•^, 95%), 2,2-azino-bis(3-ethyl- benzothiazoline-6-sulfonic acid) diammonium salt (ABTS, 98%), 2.0 M Folin–Ciocalteu phenol reagent, KCl, Na_2_HPO_4_, K_2_S_2_O_8_, NaCl, Na_2_CO_3_, and HPLC grade acetonitrile were purchased from Merck (Darmstadt, Germany); KH_2_PO_4_ from Jansen Chimica (Beerse, Belgium); 2,4,6-tripyridyl-s-triazine (TPTZ) from Fluka Chemicals (Steinheim, Switzerland). Acetone, methanol (MeOH), *n*-hexane and acetic acid were obtained from StanLab (Lublin, Poland); agricultural origin ethanol (96.6%) from Stumbras (Kaunas, Lithuania). Dimethyl sulfoxide (DMSO), *n*-butanol and ethyl acetate were purchased from Sigma-Aldrich (Saint-Quentin-Fallavier, France). Reference compounds, mangiferin and hydroxytormentic acid were purchased from Sigma Aldrich (Steinheim, Germany), and tormentic acid from ChemFaces (Wuhan, Hubei, China).

### 3.3. Preparation of Extracts

For extract preparation the ground plant samples were extracted with *n*-hexane, acetone, ethanol:water (70:30, *v*/*v*) (further 70% ethanol), and water using three different extraction methods: Soxhlet, by stirring and pressurized liquid (PLE) extraction. The obtained extracts are further marked with a lowercase letter depending on extraction method: s—single, c—consecutive, and p—pressurized. After extraction and fractionation, the solvents were removed in a rotary evaporator (Büchi, Flawil, Switzerland), while residual water was freeze-dried. The amounts of extracts and fractions were determined gravimetrically (±0.001 g). Dry extracts and fractions were stored at –20 °C for further experiments. All experiments were conducted at least in triplicate.

#### 3.3.1. Soxhlet Extraction

The sample (12 g) was placed into a cellulose thimble and extracted with *n*-hexane and acetone in a Soxhlet extractor (Behr Labor-Technik, Düsseldorf, Germany). In consecutive extractions ground plants firstly were extracted with *n*-hexane, then the residue was re-extracted with acetone.

#### 3.3.2. Stirring Assisted Extraction

For hydroethanolic (EH) extracts 5 g of plant material and residue after acetone extraction (in consecutive extraction mode) were extracted 2 times by stirring with 100 mL of 70% ethanol at room temperature for 1 h; the extracts were filtered through a Whatman No.1 filter paper and combined.

#### 3.3.3. Pressurized Liquid Extraction (PLE)

Pressurized liquid extraction (PLE) was performed in a Dionex ASE 350 system (Dionex, Sunnyvale, CA, USA) as described elsewhere [34] with some modifications. The samples consisting of 3.5 g ground plant material were mixed with diatomaceous earth (1:1) and placed in a 34 mL stainless-steel cell (2.9 cm diameter) containing a cellulose filter at the ends to avoid solid particles in the collection vial. The cells were preheated 5 min to ensure that the samples reached thermal equilibrium at 10 MPa pressure and 70 °C or 140 °C temperature before 3 extraction cycles, 5 min each (total time 15 min). Afterwards the cell was purged for 60 s with nitrogen to collect the extract in the collection vial.

#### 3.3.4. Fractionation of Crude Extract

The purified fractions of the crude hydroethanolic extract (EHs) were obtained by using liquid-liquid extraction and by precipitation at low temperature. For liquid-liquid extraction 0.5 g of dried hydroethanolic extract (EHs) was dissolved in 50 mL of distilled water and then partitioned sequentially with hexane, ethyl acetate and *n*-butanol in a separating funnel. Several successive extractions with every solvent were made, every time using 100 mL of solvent. In total 500–600 mL of each solvent were used.

The other fractions were obtained exploring the formation of precipitate at low temperature. For this, after stirring assisted extraction with 70% ethanol, ethanol was removed in a rotary evaporator and extract was stored in a refrigerator at 6 °C for 48 h. The resulting precipitate was separated from extract by centrifugation at 3651 rcf/min for 5 min and by pouring the transparent extract part to another test tube, while the precipitated fraction was removed from centrifuge tubes by adding water. After first storage the precipitated part of extract consisted of two layers: green and white-yellow, therefore they were separated by centrifugation, thus obtaining two fractions: green fraction (Sto1g) and precipitated fraction (Sto1p). The transparent extract was further stored in a refrigerator at 6 °C for 48 h and the resulting precipitate was separated by centrifugation thus obtaining the precipitated fraction (Sto2p). This procedure was repeated until the absence of precipitate. In most cases, the second storage was the last one giving precipitates, however in some cases low amount of yellow-white colors precipitate was obtained after third and fourth storages. Due to low amount of these precipitates and the same appearance as the Sto2p fraction, these precipitates were combined and analyzed together. The part of extract, which not precipitates, was assigned for transparent fraction (Tf). After fractionation organic solvents were removed in a rotary evaporator and water was freeze-dried. This experiment was performed 5 times.

### 3.4. Assessment of Antioxidant Capacity

For the analysis extracts and fractions were dissolved in DMSO and methanol (1:9, *v*/*v*) at a concentration of 10 mg/mL. Methanol was used for further dilutions needed for every individual assay. Not fully dissolved extracts were treated in the ultrasonic bath ASTRA-SON^TM^, model 9H (Heat Systems Ultrasonics, NY, USA) and filtered. The absorbances were measured with Spectronic Genesys 8 spectrophotometer (Thermo Spectronic, Rochester, NY, USA) in semi-micro cuvets (Ratiolab GmbH, Dreieich, Germany). All experiments were replicated at least 3 times.

#### 3.4.1. Total Phenolic Content (TPC)

TPC was determined by a slightly modified Folin–Ciocalteu’s method [35]. For the analysis, 150 µL of sample or MeOH (blank) were mixed with 750 µL of Folin–Ciocalteu’s reagent (previously diluted with distilled water at a ratio 1:9 (*v*/*v*)) and 600 µL of 7.5% sodium carbonate solution, left in dark for 2 h and absorbance was measured at 760 nm. The TPC was calculated from the calibration curve produced by using 150 µL gallic acid solutions (0–80 µg GA/mL ethanol) and the results were expressed as mg of GA equivalents per g of dry weight of extract or fraction (mg GAE/g DWE or DWF).

#### 3.4.2. The DPPH^•^ Scavenging Assay

The DPPH^•^ scavenging capacity of extracts was determined by the method of Brand-Williams et al. [36] with some modifications. The working solution (~90 µmol DPPH^•^/L MeOH) was prepared daily, before measurements to obtain absorption of 0.800 ± 0.03 at 517 nm. For the analysis, 500 µL of sample or MeOH (blank) were mixed with 1000 µL of working solution, left in dark for 2 h and the absorbance was read. Radical scavenging capacity was calculated from the calibration curve produced using 500 µL of Trolox solutions (0–50 µmol Trolox/L MeOH) and the results were expressed in µM of Trolox equivalents per g extract or fraction (µM TE/g DWE or DWF).

#### 3.4.3. The ABTS^•+^ Scavenging (Decolorisation) Assay

The ABTS^•+^ scavenging assay was carried out as described by Re et al. [37] with some modifications. Phosphate buffered saline (PBS) solution (75 mM/L; pH 7.4) was prepared by dissolving 8.18 g NaCl, 0.27 g, KH_2_PO_4_, 3.58, Na_2_HPO_4_·12 H_2_O and 0.15 g KCl in 1 L of distilled water. Stock ABTS^•+^ solution was prepared by mixing 50 mL ABTS (2 mM/L PBS) with 200 µL K_2_S_2_O_8_ (70 mM/L H_2_O) and keeping for 12–16 h at room temperature in the dark. Before each assay, the working solution was prepared by diluting ABTS^•+^ stock solution with PBS to obtain absorption of 0.80 ± 0.03 at 734 nm. For the analysis, 25 µL of sample or MeOH (blank) were mixed with 1500 µL of working solution, left in dark for 2 h and the absorbance was read. A series of Trolox solutions in the concentration ranges of 0–1500 µmol/L MeOH were used for the calibration and the results were expressed as in DPPH^•^ assay.

#### 3.4.4. Ferric Reducing Antioxidant Power (FRAP) Assay

The FRAP assay was carried out by the method of Benzie and Strain [38] with some modifications. FRAP reagent was prepared by mixing a solution of 10 mM TPTZ (in 40 mM HCl), 20 mM FeCl_3_∙6H_2_O and acetate buffer (300 mM, pH 3.6) at 1:1:10 (*v*/*v*/*v*). For the analysis, 50 µL of sample or MeOH (blank) were mixed with 150 µL of distilled H_2_O and 1500 µL of freshly prepared FRAP reagent, left in dark for 2 h and the absorbance was read at 593 nm. A series of Trolox solutions in the concentration ranges of 0–800 µmol/L MeOH were used for the calibration and the results were expressed as in DPPH^•^ assay.

### 3.5. Identification and Quantification of Secondary Metabolitesof A. theiformis by UPLC-MS/MS Analysis

An Acquity UPLC (Waters, Milford, MA, USA) system equipped with a binary pump, autosampler, photodiode array (PDA) detector, column manager, data station running the Compass acquisition and data software was used. Chromatographic separation of compounds was carried out on an ACQUITY BEH, C18 column (50 mm × 2.1 mm, 1.7 µm) maintained at 30 °C. The eluent system consisted of solvents A (0.4% formic acid in ultra-pure water) and B (100% acetonitrile) with a linear gradient programmed as follows: 0.0–14 min, 5% B; 15–17 min, 100% B; 18 min, 5% B. The flow rate was 0.4 mL/min, temperature of sample 10 °C and sample injection volume 1 µL. The effluents from the PDA detector were introduced directly into the quadrupole-time of flight mass spectrometer (Q-TOF) equipped with an electrospray ionization source controlled by HyStar 3.2 SR2 software (Bruker Daltonic, Bremen, Germany). All MS data were recorded in ESI negative ionization mode in a range of 80–1200 m/z, the capillary voltage was maintained at +4000 V. Nitrogen was used as a nebulizer gas at 2.5 bar and drying gas at flow rate of 10 L/min. The peaks were identified by comparing their retention times and parent ions with external standards, references, and commercial databases.

Selected compounds were quantified using an Acquity UPLC^TM^ H-Class equipped with Xevo TQ-S tandem quadrupole mass spectrometer (Waters, Milford, MA) operating in negative electrospray ionization (ESI) mode, capillary voltage was set to 1500 V, cone voltage—20 V, source offset—50 V. Desolvation temperature was 500 °C, desolvation gas flow—1000 L/h, cone gas flow—150 L/h and nebulizer pressure was set to 7 bar. Chromatographic separation was performed using the same column and solvents as described above with a linear gradient programmed as: 0.0–9 min, 100% A; 9–10 min, 100% B; 10–14 min, 100% A. The flow rate was 0.4 mL/min and sample injection volume was 2 µL. MS detection was achieved in the multiple reaction monitoring (MRM) mode. MRM condition for mangiferin screening: precussor ion (*m*/*z*) was set to 421.08, product ion (*m*/*z*)—to 330.92, dwell time—0.002 s, cone voltage—64 V, collision energy—20 V. In the case of tormentic acid precussor ion (*m*/*z*) was set to 487.34, product ion—to 437.12, dwell time—0.05 s, cone voltage—80 V, collision energy—36 V, while in case of hydroxytormentic acid precussor ion (*m*/*z*) was set to 503.34, product ion—to 453.11, dwell time—0.05 s, cone voltage—94 V, and collision energy—36 V. MassLynx 4.1 software (Waters, Milford, USA) was used for instrument control and data collection. All samples were run in triplicates. The concentrations of secondary metabolites were calculated from calibration curves prepared using concentrations of 0.02–40 µg/mL of different standard compounds: mangiferin (y = 33.948x + 83711; R^2^ = 0.9949), tormentic acid (y = 5.0611x + 270.28; R^2^ = 0.9917), hydroxytormentic acid (y = 4.0672x + 5823; R^2^ = 0.9916). The results were expressed in the dry weight of extract (DWE) or fraction (DWF). The amount of quantified compounds in extracts was also recalculated to the dry weight of the whole plant material (DWP). For the determination of fragmentation patterns of some compounds scan wave DS (daughter scan) function was applied.

### 3.6. Statistical Data Analysis

Statistical analysis was performed using GraphPad Prism software (version 5.0; GraphPad software, La Jola, CA, USA). Results were subjected to analysis of variance (one-way ANOVA) and differences between means were calculated using Tukey’s multiple comparison test. Differences were considered to be significant when *P*-values were below 0.05 (*p* < 0.05). All data were expressed as mean ± standard deviation (SD). Correlation coefficients for mangiferin content/TPC and antioxidant activity assays were calculated using Pearson’s correlation coefficient using MS Excel 2010.

## 4. Conclusions

Application of various extraction/fractionation techniques and solvents as well as single-step and consecutive-extraction procedures to *A. theiformis* leaves demonstrated strong antioxidant capacity (equivalent to synthetic antioxidant Trolox) of polar plant fractions isolated by ethanol/water mixture and acetone. It was proved that *A. theiformis* leaves are excellent sources of bioactive phytochemical mangiferin and tormentic and hydroxytormentic acids and their derivatives were other abundant constituents. The ethanol/water mixture was the most efficient solvent for recovery of antioxidants, including mangiferin, in all of the applied procedures. Therefore, it was further fractionated with various solvents and by precipitation at low temperature: *n*-butanol and two precipitated fractions (Sto1p and Sto2p) demonstrated very high antioxidant capacity (comparable to the pure mangiferin). Strong correlation between mangiferin concentration and antioxidant capacity of fractions was observed. However, some fractions were even stronger radical scavengers than pure mangiferin. This finding suggests that other constituents of *A. theiformis* can also contribute to high antioxidant potential by themselves and/or by exerting synergistic effects. To the best of our knowledge, this study is the first comprehensive evaluation of *A. theiformis* antioxidant potential and composition of secondary metabolites; quantification of mangiferin, tormentic and hydroxytormentic acids in extracts and fractions has not been reported previously. Consequently, this study reveals that the *A. theiformis* extracts and its fractions may be promising health beneficial ingredients for nutraceuticals, functional foods, cosmetics, and even pharmaceuticals.

## Figures and Tables

**Table 1:** Yield, total phenolic content (TPC) (in mg GAE/g dry weight of extract (DWE)) and antioxidant capacity (in µM TE/g DWE) of *A. theiformis* extracts obtained by various solvents and extraction methods and recovery of antioxidants by various extraction processes (in mg GAE/g dry weight of the initial plant material (DWP) or µM TE/g DWP).

Solvent, Procedure	Yield, %		TPC		DPPH^•^		ABTS^•+^		FRAP	
	DWR	DWP	DWE	DWP	DWE	DWP	DWE	DWP	DWE	DWP
*Whole material, 1-step*										
HE (*SE*)	2.62 ± 0.04 ^a^	2.62 ^a^	-	-	-	-	-	-	-	-
ACs (*SE*)	10.97 ± 0.79 ^e^	11.0 ^e^	247 ± 13.0 ^d^	27.1 ^c^	2451 ± 96.8 ^c^	269 ^c^	4533 ± 77.6 ^g^	497 ^c^	3360 ± 176 ^d^	369 ^d^
EHs (*SAE*)	34.07 ± 0.66 ^j^	34.1 ^g^	330 ± 11.9 ^f^	112 ^g^	3267 ± 81.5 ^e^	1113 ^f^	4595 ± 111 ^f,g^	1566 ^f^	4473 ± 129 ^f^	1524 ^f^
*Consecutive*										
HE (*SE*)	2.62 ± 0.04 ^a^	2.62 ^a^	-	-	-	-	-	-	-	-
ACc (*SE*)	9.40 ± 0.37 ^d^	9.15 ^d^	275 ± 14.0 ^e^	25.1 ^c^	2902 ± 46.8 ^d^	266 ^c^	5091 ± 118 ^h^	466 ^c^	3905 ± 45.1 ^e^	367 ^d^
EHc (*SAE*)	28.79 ± 0.33 ^i^	25.3 ^f^	317 ± 6.76 ^f^	80.2 ^e^	3121 ± 93.8 ^e^	791 ^e^	4313 ± 54.2 ^e,f^	1093 ^d^	3388 ± 117 ^d^	858 ^e^
**∑**		**37.1**		**105**		**1057**		**1559**		**1225**
HEp_70_ (*PLE*)	2.47 ± 0.23 ^a^	2.47 ^a^	-	-	-	-	-	-	-	-
ACp_70_ (*PLE*)	6.65 ± 0.02 ^c^	6.49 ^c^	172 ± 5.00 ^c^	11.2 ^b^	1884 ± 51.1 ^b^	122 ^a^	3025 ± 76.8 ^d^	196 ^b^	2160 ± 79.5 ^b^	144 ^b^
EHp_70_ (*PLE*)	27.86 ± 0.36 ^h^	25.4 ^f^	322 ± 23.4 ^f^	88.5 ^f^	2867 ± 34.4 ^d^	824 ^e^	4598 ± 146 ^g^	1166 ^e^	3504 ± 102 ^d,e^	889 ^e^
Wp_70_ (*PLE*)	13.45 ± 0.29 ^f^	8.83 ^d^	138 ± 3.65 ^b^	12.2 ^b^	2314 ± 89.9 ^c^	204 ^b^	2378 ± 30.9 ^b^	210 ^b^	2317 ± 34.1 ^c^	205 ^c^
**∑**		**43.2**		**112**		**1053**		**1572**		**1238**
HEp_140_ (*PLE*)	3.59 ± 0.13 ^b^	3.59 ^b^	-	-	-	-	-	-	-	-
ACp_140_ (*PLE*)	6.72 ± 0.01 ^c^	6.48 ^c^	154 ± 4.05 ^c^	10.0 ^b^	1748 ± 26.4 ^b^	113 ^a^	2683 ± 87.4 ^c^	174 ^b^	2119 ± 28.7 ^b^	142 ^b^
EHp_140_ (*PLE*)	28.80 ± 0.13 ^i^	25.9 ^f^	291 ± 1.98 ^e^	75.4 ^d^	2837 ± 76.4 ^d^	735 ^d^	4165 ± 138 ^e^	1079 ^d^	3362 ± 97.3 ^d^	871 ^e^
Wp_140_ (*PLE*)	14.65 ± 0.10 ^g^	9.38 ^d^	7.59 ± 0.55 ^a^	0.71 ^a^	1395 ± 111 ^a^	131 ^a^	107 ± 2.89 ^a^	10.1 ^a^	290 ± 26.3 ^a^	30.7 ^a^
**∑**		**45.4**		**86**		**979**		**1263**		**1044**

SE—Soxhlet extraction, SAE—Stirring assisted extraction, PLE—Pressurized liquid extraction. Extracts: HE—hexane, AC—acetone, EH—hydroethanol, W—water. Values represented as mean ± standard deviation (*n* = 3); columns with different letters differ significantly for Tukey’s test at *p* < 0.05; DWR—dry weight residue; DWE—dry weight extract; DWP—dry weight initial plant.

**Table 2:** Yield, total phenolic content (TPC) (in mg GAE/g DWE or DWF), and antioxidant capacity (in µM TE/g DWE or DWF) of crude hydroethanolic extract and its fractions.

Product	Yield, %	TPC	DPPH^•^	ABTS^•+^	FRAP
Crude hydroethanolic extract (EHs)	34.07 ± 0.66	362 ± 6.28 ^e^	3267 ± 81.5 ^f^	4595 ± 111 ^e^	4473 ± 129 ^e^
Fractions obtained using liquid-liquid extraction					
Hexane	14.2 ± 0.3	42.4 ± 0.62 ^a^	302 ± 5.98 ^a^	511 ± 21.8 ^a^	461 ± 17.1 ^a^
Ethyl acetate	17.0 ± 0.81	208 ± 2.77 ^b^	1340 ± 26.3 ^b^	2106 ± 46.0 ^b^	2030 ± 104 ^c^
*n*-Butanol	48.0 ± 0.91	423 ± 4.79 ^f^	3496 ± 28.4 ^g^	4555 ± 26.2 ^e^	4818 ± 85.5 ^g^
Water	17.7 ± 2.41	257 ± 4.78 ^c^	1969 ± 92.4 ^d^	2589 ± 14.1 ^c^	1476 ± 68.8 ^b^
Fractions obtained by cooling at 6 °C					
Sto1g	2.06 ± 8.23	201 ± 3.68 ^b^	1568 ± 51.5 ^c^	1946 ± 47.5 ^b^	1857 ± 109 ^c^
Sto1p	14.14 ± 29.12	491 ± 16.6 ^g^	4333 ± 21.0 ^h^	6878 ± 47.8 ^f^	4298 ± 129 ^e^
Sto2p	2.06 ± 19.54	599 ± 4.40 ^h^	4816 ± 62.3 ^i^	7951 ± 360 ^g^	5582 ± 87.7 ^f^
Tf	63.7	300 ± 3.56 ^d^	2359 ± 39.7 ^e^	4054 ± 175 ^d^	2656 ± 54.6 ^d^
Mangiferin	-	668 ± 8.58 ^i^	4282 ± 246 ^h^	7227 ± 226 ^f^	6750 ± 2.13 ^h^

Sto1g—green fraction (storage 1), Sto1p—precipitated fraction (storage 1), Sto2p—precipitated fraction (storage 2), Tf—transparent fraction. DWF—dry weight of fraction. Values represented as mean ± standard deviation (*n* = 3); columns with different letters differ significantly for Tukey’s test at *p* < 0.05.

**Table 3:** Chemical profile of *A. theiformis* EHs extract and its fractions analysed by UPLC-QTOF-MS.

Peak No.	RT	Compound	Molecular Formula	*m*/*z*	
				[M − H]^−^	MS Fragments
1.	0.35	Unknown, [M − H]^−^ similar to fructose	C_6_H_12_O_6_	179.0563	-
2.	1.70	Unknown, [M − H]^−^ similar to iriflophenone-3-C-β-d-glucopyranoside	C_19_H_20_O_10_	407.0984	-
3.	1.85	Mangiferin **	C_19_H_18_O_11_	421.0777	-
4.	2.75	Unknown saponin *	C_37_H_60_O_14_	727.3910	-
5.	3.20	Hydroxytormentic acid derivative *	C_37_H_60_O_13_	711.3961	503.3371 [HTA − H]^−^
6.	3.60	Tormentic acid derivative *	C_37_H_60_O_12_	695.4012	649.3949, 487.3427
					[TA − H]^−^
7a.	3.61	23-Hydroxytormentic acid isomer *	C_30_H_48_O_6_	503.3372	-
7b.	4.73	23-Hydroxytormentic acid **	C_30_H_48_O_6_	503.3372	-
7c.	5.14	23-Hydroxytormentic acid isomer *	C_30_H_48_O_6_	503.3372	-
8.	4.45	Unknown	C_16_H_28_O_6_	315.1813	-
9a.	4.34	Tormentic acid isomer *	C_30_H_48_O_5_	487.3429	-
9b.	5.60	Tormentic acid **	C_30_H_48_O_5_	487.3429	-
9c.	5.74	Tormentic acid isomer *	C_30_H_48_O_5_	487.3429	-
10.	5.85	Unknown	C_18_H_30_O_3_	293.2027	-
11.	6.35	Unknown, [M − H]^−^ similar to maslinic/corosolic acid	C_30_H_48_O_4_	471.3480	-
12.	6.40	Unknown	C_18_H_32_O_3_	295.2279	-
13.	4.13	Unknown	C_30_H_48_O_7_	520.7039	-
14.	5.90	Unknown, [M − H]^−^ similar to quillaic acid	C_30_H_46_O_5_	485.3272	-

** Confirmed by a standard; * tentatively identified.

**Table 4:** Quantification of secondary metabolites present in the extracts of *A. theiformis* mg/g DWE or mg/g DWP.

Sample	Mangiferin		Hydroxytormentic acid RT = 4.73 (7b)		Hydroxytormentic acid isomer * RT = 3.61 (7a)		Hydroxytormentic acid isomer * RT = 5.14 (7c)		Tormentic acid RT = 5.60 (9b)		Tormentic acid isomer * RT = 4.34 (9a)		Tormentic acid isomer * RT = 5.74 (9c)	
	DWE	DWP	DWE	DWP	DWE	DWP	DWE	DWP	DWE	DWP	DWE	DWP	DWE	DWP
*1-step*														
ACs (*SE*)	38.10 ± 0.11 ^b^	4.18 ^b^	2.71 ± 0.02 ^e^	0.30 ^c^	3.19 ± 0.03 ^d^	0.35 ^d^	0.20 ± 0.00 ^b^	0.02 ^a^	0.16 ± 0.01 ^b^	0.02 ^b^	0.21 ± 0.01 ^b^	0.02 ^c^	3.70 ± 0.03 ^d^	0.40 ^d^
EHs (*SAE*)	104.6 ± 3.99 ^f^	35.6 ^f^	1.26 ± 0.04 ^d^	0.43 ^e^	1.70 ± 0.21 ^c^	0.58 ^e^	0.01 ± 0.00 ^a^	tr	0.15 ± 0.00 ^a^	0.05 ^c^	0.24 ± 0.00 ^c^	0.08 ^d^	1.72 ± 0.12 ^c^	0.59 ^e^
*Consecutive*														
ACc (*SE*)	43.03 ± 0.05 ^b,c^	3.94 ^b^	3.01 ± 0.05 ^f^	0.28 ^c^	3.36 ± 0.06 ^d^	0.31 ^b,c,d^	0.22 ± 0.01 ^b^	0.02 ^a^	0.18 ± 0.01 ^b^	0.02 ^b^	0.21 ± 0.00 ^b^	0.02 ^a^	3.45 ±0.06 ^d^	0.32 ^c^
EHc (*SAE*)	101.0 ± 0.01 ^f^	25.6 ^e^	0.34 ± 0.01 ^c^	0.09 ^b^	0.86 ± 0.01 ^b^	0.22 ^b,c^	nd	nd	nd	nd	0.01 ± 0.00 ^a^	tr	1.03 ± 0.01 ^b^	0.26 ^b,c^
**∑**		**29.5**		**0.37**		**0.53**		**0.02**		**0.02**		**0.02**		**0.58**
ACp_70_ (*PLE*)	48.64 ± 0.24^c^	3.16 ^b^	3.94 ± 0.02 ^g^	0.26 ^c^	3.72 ± 0.03 ^e^	0.24 ^c^	0.31 ± 0.00 ^c^	0.02 ^a^	0.24 ± 0.00 ^c^	0.02 ^b^	0.28 ± 0.00 ^d^	0.02 ^a^	4.28 ± 0.12 ^e^	0.28 ^b,c^
EHp_70_ (*PLE*)	90.46 ± 3.25 ^e^	23.0 ^d^	0.31 ± 0.00 ^b^	0.08 ^b^	0.95 ± 0.01 ^b^	0.24 ^c^	nd	nd	nd	nd	0.02 ± 0.00 ^a^	tr	0.99 ± 0.00 ^b^	0.25 ^b^
Wp_70_ (*PLE*)	87.13 ± 0.02 ^e^	7.69 ^c^	0.04 ± 0.00 ^a^	d	0.27 ± 0.00 ^a^	0.02 ^a^	nd	nd	nd	nd	Tr	tr	0.52 ± 0.00 ^a^	0.05 ^a^
**∑**		**33.9**		**0.34**		**0.50**		**0.02**		**0.02**		**0.02**		**0.58**
ACp_140_ (*PLE*)	72.20 ± 2.64 ^d^	4.68 ^b^	5.63 ± 0.08 ^h^	0.36 ^d^	3.25 ± 0.01 ^d^	0.21 ^b,c^	0.32 ± 0.01 ^c^	0.02 ^b^	0.15 ± 0.00 ^a^	0.01 ^a^	0.24 ± 0.01 ^c^	0.02 ^b^	4.05 ± 0.11 ^e^	0.26 ^b,c^
EHp_140_ (*PLE*)	86.46 ± 0.06 ^e^	22.4 ^d^	0.14 ± 0.00 ^a^	0.04 ^a^	0.56 ± 0.01 ^a^	0.14 ^b^	nd	nd	nd	nd	tr	tr	tr	tr
Wp_140_ (*PLE*)	12.18 ± 0.30 ^a^	1.14 ^a^	tr	tr	tr	tr	nd	nd	nd	nd	nd	nd	tr	tr
**∑**		**28.2**		**0.40**		**0.35**		**0.02**		**0.01**		**0.02**		**0.26**

SE—Soxhlet extraction, SAE—Stirring assisted extraction, PLE—Pressurized liquid extraction. nd—not detected, tr—trace. Tormentic acid (C_30_H_48_O_5_); mangiferin (C_19_H_18_O_11_); 23-hydroxytormentic acid (C_30_H_48_O_6_). * expressed as tormentic or hydroxytormentic acids equivalents. Values represented as mean ± standard deviation (*n* = 3); columns with different letters differ significantly for Tukey’s test at *p* < 0.05.

**Table 5:** Quantification of selected secondary metabolites present in the fractions of *A. theiformis* mg/g DWF.

Fraction	Mangiferin	Hydroxytormentic acid RT = 4.73 (7b)	Hydroxytormentic acid isomer * RT = 3.61 (7a)	Hydroxytormentic acid isomer * RT = 5.14 (7c)	Tormentic acid RT = 5.60 (9b)	Tormentic acid isomer * RT = 4.34 (9a)	Tormentic acid isomer * RT = 5.74 (9c)
Ethyl acetate	191.7 ± 3.21 ^c^	4.02 ± 0.22 ^b^	5.83 ± 0.06 ^d^	tr	tr	tr	8.51 ± 0.01 ^b^
n-butanol	416.3 ± 3.77 ^d^	nd	1.30 ± 0.06 ^b^	nd	nd	nd	tr
Water	12.57 ± 0.10 ^a^	tr	tr	nd	nd	nd	tr
Sto1g	152.5 ± 7.11 ^b^	6.31 ± 0.01 ^c^	0.59 ± 0.03 ^a^	0.05 ± 0.00 ^a^	tr	nd	1.00 ± 0.01 ^a^
Sto1p	459.7 ± 11.2 ^e^	4.59 ± 0.28 ^b^	nd	tr	tr	nd	9.98 ± 0.10 ^c^
Sto2p	557.0 ± 15.4 ^f^	0.10 ± 0.00 ^a^	tr	tr	nd	nd	8.30 ± 0.09 ^b^
Tf	182.6 ± 5.97 ^c^	tr	3.19 ± 0.03 ^c^	nd	nd	tr	tr

Nd—not detected, tr—trace. Tormentic acid (C_30_H_48_O_5_); mangiferin (C_19_H_18_O_11_); 23-hydroxytormentic acid (C_30_H_48_O_6_). * expressed as tormentic or hydroxytormentic acids equivalents. Values represented as mean ± standard deviation (*n* = 3); columns with different letters differ significantly for Tukey’s test at *p* < 0.05.
